# Molecular Evolution of Aralkylamine *N*-Acetyltransferase in Fish: A Genomic Survey

**DOI:** 10.3390/ijms17010051

**Published:** 2015-12-31

**Authors:** Jia Li, Xinxin You, Chao Bian, Hui Yu, Steven L. Coon, Qiong Shi

**Affiliations:** 1 BGI Education Center, University of Chinese Academy of Sciences, Shenzhen 518083, China; lijia1@genomics.cn (J.L.); yuhui@genomics.cn (H.Y.); 2 Shenzhen Key Lab of Marine Genomics, Guangdong Provincial Key Lab of Molecular Breeding in Marine Economic Animals, BGI, Shenzhen 518083, China; youxinxin@genomics.cn (X.Y.); bianchao@genomics.cn (C.B.); 3 BGI-Zhenjiang Institute of Hydrobiology, Zhenjiang 212000, China; 4 Molecular Genomics Laboratory, National Institutes of Health, Bethesda, MD 20892, USA

**Keywords:** aralkylamine *N*-acetyltransferase, phylogenetic analysis, synteny, molecular evolution, Whole-Genome Duplication (WGD), gene loss

## Abstract

All living organisms synchronize biological functions with environmental changes; melatonin plays a vital role in regulating daily and seasonal variations. Due to rhythmic activity of the timezyme aralkylamine *N*-acetyltransferase (AANAT), the blood level of melatonin increases at night and decreases during daytime. Whereas other vertebrates have a single form of AANAT, bony fishes possess various isoforms of *aanat* genes, though the reasons are still unclear. Here, we have taken advantage of multiple unpublished teleost *aanat* sequences to explore and expand our understanding of the molecular evolution of *aanat* in fish. Our results confirm that two rounds of whole-genome duplication (WGD) led to the existence of three fish isoforms of *aanat*, *i.e*., *aanat1a*, *aanat1b*, and *aanat2*; in addition, gene loss led to the absence of some forms from certain special fish species. Furthermore, we suggest the different roles of two *aanat1s* in amphibious mudskippers, and speculate that the loss of *aanat1a*, may be related to terrestrial vision change. Several important sites of AANAT proteins and regulatory elements of *aanat* genes were analyzed for structural comparison and functional forecasting, respectively, which provides insights into the molecular evolution of the differences between AANAT1 and AANAT2.

## 1. Introduction

Light stimulus is a key factor for maintaining the physiological balance of fish. The day/night variation may exert an influence over their entire lifetime. Several organs participate in receiving external light signals. In all vertebrates, the retina is the main photoreceptive organ for reception of photic input [1]. In contrast to mammals, the pineal gland of lower vertebrates, such as fish, can also detect changes in environmental light levels. The primary function of the pineal gland is to produce and release melatonin in response to circadian and lighting stimulation. Circulating melatonin levels are much higher at night than during daytime in essentially all vertebrates. The difference in blood melatonin levels between daytime and night provides information to the rest of the organism regarding the time of day and duration of the night period. Melatonin can regulate the endocrine activities through binding to its receptors throughout the fish body [2], hence it is considered a key component of the circadian system. For instance, we demonstrated that melatonin regulates reproduction in blue-spotted mudskipper (*Boleophthalmus pectinirostris*, a semilunar spawning fish) by combining with receptors of the hypothalamic-pituitary-gonad (HPG) axis or directly acting on gonadal tissues [3]. Melatonin can also influence the appetite of fish because of melatonin binding sites in gastrointestinal tract [4,5,6]. Melatonin is thought to influence wide-ranging physiologies including reproduction, sleep, the immune system, and so on [7,8,9,10].

Melatonin is synthesized from tryptophan (Trp) through four enzyme-catalyzed reactions [11]. The first step converts Trp to 5-hydroxytryptophan (5-HTrp) by tryptophan hydroxylase (TPH, EC 1.14.16.4). The subsequent reaction converts 5-HTrp to 5-hydroxytryptamine (5-HT, serotonin) through the catalysis by of aromatic-l-amino-acid decarboxylase (AAAD, EC 4.1.1.28). The third product is *N*-acetylserotonin (NAS), formed by acetylation of 5-HT by aralkylamine *N*-acetyltransferase (AANAT, EC 2.3.1.87). The last reaction is catalyzed by acetylserotonin-*O*-methyltransferase (ASMT, EC 2.1.1.4) to generate melatonin.

AANAT, also known as serotonin *N*-acetyltransferase (SNAT), is the penultimate enzyme in the synthesis of melatonin [12,13]. As a result of catalysis by AANAT, 5-HT receives an acetyl group from acetyl CoA (AcCoA) to become NAS. The large daily rhythms in melatonin production in the pineal gland are driven by changes in AANAT activity; these changes may be due to both regulation at the transcriptional level and through post-translational modifications. The last enzyme in melatonin synthesis, ASMT, usually shows little day/night variation, but may only provide an upper limit, or cap, on the maximum amount of melatonin produced. Thus, to better understand control of melatonin synthesis in fish, we sought to explore the evolution of *aanat* and to explain the reason for the existence of multiple *aanat* isoforms in fish.

The vertebrate-type of AANAT evolved from a more-primitive non-vertebrate-type of AANAT [14]. The primitive AANAT is widely-known from organisms evolutionarily more ancient than Agnathans, including amphioxus [15]. During early vertebrate evolution, this gene was duplicated and one of the paralogs apparently diverged to become the vertebrate-type AANAT. The non-vertebrate-type was lost from almost all vertebrate lineages, but the two forms coexist in certain chondrichthyes (specifically the elephant shark, *Callorhinchus milii* [14]). In teleosts and tetrapods, only the vertebrate-type exists, and the multiple forms of this gene are the subject of this report. Prior to the divergence of Gnathostomes, the vertebrate-type AANAT was duplicated, but only one of these duplicates was retained in the tetrapods. In teleosts, the two isoforms were retained and are called *aanat1* and *aanat2*. Subsequently *aanat1* was again duplicated to yield *aanat1a* and *aanat1b*. Throughout teleost evolution, the timing of these duplication, and selective losses of isoforms, have influenced regulation of melatonin production. Additionally, numerous teleost species are tetraploid, adding further complexity.

In addition to the presence or absence of *aanat* isoforms, levels of expression can influence melatonin production. Several important DNA regulatory elements can influence the expression of *aanat*. Specifically, photoreceptor conserved element (PCE, TAATT/C) is the most common regulatory element of *aanat*. In zebrafish, PCE plays a role in pineal-specific expression of *aanat2* [16]*.* Also, E-box (CACGTG) and E-box-like element (CATGTG or TACGTG) can strengthen pineal-specific expression of *aanat2* and suppress non-pineal gland expression [17,18]*.* Meanwhile, some circadian genes can also direct regulation through the E-box [19]. The *cis*-acting regulatory element (CAATC), which is also called pineal expression-promoting element (PIPE), directs pineal-specific gene expression in zebrafish [20].

**Figure 1:**
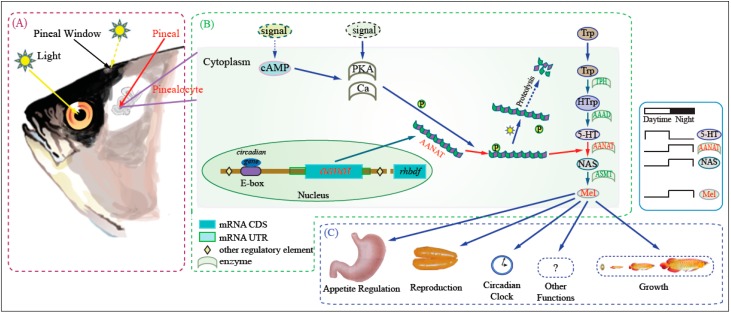
Outline of melatonin biosynthesis and function. Amphibians, reptiles, and fishes can detect light by photoreceptor cells of the retinae and pinealocytes of the pineal glands. (**A**) Photic detecting and transforming system includes the retina photoreceptor cells and the pinealocytes; (**B**) Melatonin synthesis pathway is summarized in the frame; (**C**) Melatonin performs its various biological functions in different tissues and at different growth/development phases through binding to its receptors. In mammals the photoreceptive role of the pineal gland is lost.

In general, the synthesis of melatonin in fish depends on output from the circadian clock and input from external signals (Figure 1), primarily through effects on AANAT activity [21,22]. The circadian clock directly drives transcription of *aanat2* at night. The translated AANAT2 is phosphorylated by cyclic-AMP-dependent activation of protein kinase A (PKA). Phosphorylated AANAT2 (pAANAT2) binds to 14-3-3 proteins which protects it from proteasomal proteolysis. This results in elevated AANAT2 activity and rapid melatonin synthesis. External input, for instance light during the daytime, decreases the cyclic-AMP-dependent activation of protein kinase A (PKA) as well as cellular calcium levels; both effects lead to decreased phosphorylation of AANAT and subsequent destruction by the proteasome. This decreases melatonin production [23].

Melatonin is transported through the circulatory system and binds to melatonin receptors in different organs (for example ovary) to exert its effects. Thus, melatonin, synthesized in the pineal gland, plays an important endocrine role in regulating physiological activities [24]. However, melatonin secreted by the retina may play a paracrine role [25], and *aanat1* may acetylate retinal dopamine [25,26,27]. Furthermore, *aanat* gene expression is not restricted to the retina and the pineal gland. In some organisms, like goldfish, low levels of AANAT expression can also be detected in other organs, like skin, the gastrointestinal tract and brain [28,29].

With development of the next-generation sequencing technologies, the entire genomic sequences of many fish species have become publicly available. In addition, we have access to, and include here, many unpublished fish *aanat* gene and protein sequences, including amphibious teleost fish (mudskipper), cave-restricted or eyeless fish (*Sinocyclocheilus anshuiensis*), tetraploid fishes (Amazon molly and *Sinocyclocheilus* spp.) and migratory fishes (rainbow trout and salmon), which have been derived from whole genome sequencing. This allows us to determine the presence or absence of *aanat* isoforms, as well as sequence differences (both coding and non-coding) across species. In this report we have analyzed the regulatory regions of *aanat* genes, compared the protein sequence differences in three AANAT isoforms (AANAT1a, AANAT1b, and AANAT2), constructed phylogenetic trees and completed synteny analysis. Finally, we discussed the molecular and functional evolution of AANAT in fish.

## 2. Results

### 2.1. Variation of Aanat Copy Number in Different Species

A total of 84 *aanat* sequences were derived from 37 vertebrate species; these sequences and their encoded proteins were used for our data analysis. Sequences for 25 species, including seven mammal species, one bird species, two reptile species, one amphibian species and 14 fish species, were downloaded from the NCBI and Ensembl databases (related accession numbers are given in Table 1). Sequences extracted from another 12 fish species whose unpublished genomes were sequenced by us, or our collaborators, were included (see details in Table 2).

**Table 1:** The accession numbers for known *aanat* sequences.

Class	Common Name	Species Name	*aanat*	Location	Accession Number		Source
					Nucleotide Sequence	Protein Sequence	
**Mammalis**	Human	*Homo sapiens*	1	chromosome: 17	NP_001160051.1	EAW89400.1	NCBI
	House mouse	*Mus musculus*	1	chromosome: 11	NM_009591.3	EDL34585.1	NCBI
	Chimpanzee	*Pan troglodytes*	1	chromosome: 17	AY665273.2	AAV74311.2	NCBI
	Sheep	*Ovis aries*	1	chromosome: 11	NM_001009461.1	NP_001009461.1	NCBI
	Minke whale	*Balaenoptera acutorostrata scammoni*	1	chromosome: Un	XM_007185968.1	XP_007186030.1	NCBI
	Platypus	*Ornithorhynchus anatinus*	1	Ultra430:237283–238471: −1	ENSOANG00000011565	ENSOANT00000018326	Ensembl
	Panda	*Ailuropoda melanoleuca*	1	GL192703.1:1467291–1468587: 1	ENSAMEG00000010321	ENSAMET00000011309	Ensembl
**Aves Reptilia**	Rock pigeon	*Columba livia*	1	chromosome: Un	XM_005503150	XP_005503207.1	NCBI
	Green sea turtle	*Chelonia mydas*	1	chromosome: Un	XM_007072534.1	EMP23774	NCBI
	Anole lizard	*Anolis carolinensis*	1	chromosome: 2	XM_003217189.2	XP_003217237.1	NCBI
**Amphibia**	Western clawed frog	*Xenopus laevis*	1	chromosome: Un	XM_002935933.2	XP_002935979.1	NCBI
**Actinopterygii**	Zebrafish	*Danio rerio*	1	chromosome: 6	BC059448.1	AAH59448.1	NCBI
			2	chromosome: 3	NM_131411.2	NP_571486.1	NCBI
	Mexican tetra	*Astyanax mexicanus*	1	KB882152.1:1429796–1431775: −1	XM_007252167.1	XP_007252229.1	NCBI
			2	KB872051.1:338907-348651: −1	ENSAMXG00000016495	ENSAMXT00000016988	Ensembl
	Gilthead seabream	*Sparus aurata*	1	–	AY533402.1	AAT02159.1	NCBI
			2	–	AY533403.2	AAT02160.2	NCBI
	European seabass	*Dicentrarchus labrax*	1a	–	EU378922.1	ACB13284.1	NCBI
			1b	–	EU378923.1	ACB13285.1	NCBI
	Atlantic cod	*Gadus morhua*	1	GeneScaffold 1449:283672–285101: −1	ENSGMOG00000010804	ENSGMOT00000011867	Ensembl
			2	GeneScaffold 1876:387650–388957: −1	ENSGMOG00000004277	ENSGMOT00000004660	Ensembl
	Medaka	*Oryzias latipes*	1a	chromosome: 1	NM_001104832.1	NP_001098302.1	NCBI
			1b	chromosome: 8	NM_001104860.1	NP_001098330.1	NCBI
			2	chromosome: 8	AB248276.1	BAE78762.1	NCBI
	Fugu	*Takifugu rubripes*	1a	scaffold 2391:5485–6279: −1	ENSTRUG00000017502	ENSTRUT00000045022	Ensembl
			1b	scaffold 65:466946-468376: −1	ENSTRUG00000009927	ENSTRUT00000025045	Ensembl
			2	scaffold15:451903–452747: −1	ENSTRUG00000011652	ENSTRUT00000029563	Ensembl
	Tilapia	*Oreochromis niloticus*	1a	GL831193.1:3303726–3305445: 1	ENSONIG00000018253	ENSONIT00000023007	Ensembl
			1b	Un random:3751825–3761582: 1	ENSTNIG00000004312	ENSTNIT00000007096	Ensembl
			2	3:12195390–12196528: −1	ENSTNIG00000018581	ENSONIT00000001810	Ensembl
	Tetraodon	*Tetraodon nigroviridis*	1a	Un random:26969504–26970941: −1	ENSTNIG00000017516	ENSTNIT00000020893	Ensembl
			1b	Un random:3751825–3761582: 1	ENSTNIG00000004312	ENSTNIT00000007096	Ensembl
			2	3:12195390–12196528: −1	ENSTNIG00000018581	ENSTNIT00000021990	Ensembl
	Amazon molly ^1^	*Poecilia formosa*	1a	KI519952.1:480412–483140: −1	ENSPFOG00000012646	ENSPFOT00000012663	Ensembl
			1b	KI519724.1:1037528–1042207: 1	ENSPFOG00000003689	ENSPFOT00000003582	Ensembl
			2a	KI519725.1:742206-743398: −1	ENSPFOG00000010360	ENSPFOT00000010350	Ensembl
			2b	KI519725.1:771388–772351:-1	ENSPFOG00000010492	ENSPFOT00000010477	Ensembl
	Platyfish	*Xiphophorus maculatus*	1a	JH556678.1:1270978–1287717: 1	ENSXMAG00000006015	ENSXMAT00000006036	Ensembl
			1b	JH557102.1:45038–49228: 1	ENSXMAG00000008291	ENSXMAT00000008323	Ensembl
			2	JH556906.1:38852–39807: 1	ENSXMAG00000000128	ENSXMAT00000000129	Ensembl
	Stickleback ^2^	*Gasterosteus aculeatus*	1a	groupIX:16205994–16208003:-1	ENSGACG00000019276	ENSGACT00000025530	Ensembl
			1b	groupXI:3766738–3767762: −1	ENSGACG00000007070	ENSGACT00000009381	Ensembl
			2	groupXI:13702853–13703781: −1	ENSGACG00000013957	ENSGACT00000018464	Ensembl
**Chondrichthyes arcopterygii**	Elephant shark	*Callorhinchus milii*	1	chromosome: Un	NM_001292772.1	NP_001279701.1	NCBI
	Coelacanth	*Latimeria chalumnae*	1	chromosome: Un	XM_005993207.1	XP_005993269.1	NCBI

^1^ Tetraploid; ^2^ As a genome reference sequence for synteny analysis.

**Table 2:** The copy number of *aanat* genes from the fishes sequenced by our lab or collaborators.

Class	Common Name	Species Name	Total Number	*aanat1a*	*aanat1b*	*aanat2*
Actinopterygii	Giant-fin mudskipper/PM ^1^	*Periophthalmus magnuspinnatus*	2	–	1	1
	Blue-spotted mudskipper/BP ^1^	*Boleophthalmus pectinirosris*	3	1	1	1
	Malabar grouper	*Epinephelus malabaricus*	3	1	1	1
	Half-smooth tongue sole	*Cynoglossus semilaevis*	3	1	1	1
	Hedgehog seahorse	*Hippocampus spinosissimus*	3	1	1	1
	Atlantic salmon ^2^	*Salmo salar*	5	1	2	2
	Rainbow trout ^2^	*Oncorhynchus mykiss*	5	1	2	2
	Northern pike	*Esox lucius*	3	1	1	1
	Golden-line fishes (SIA) ^2^	*Sinocyclocheilus anshuiensis*	3	1	–	2
	(SIG) ^2^	*Sinocyclocheilus graham*	3	1	–	2
	(SIR) ^2^	*Sinocyclooheilus rhinocerous*	3	1	–	2
	Asian arowana	*Scleropages formosus*	3	1	1	1

^1^ Amphibious fishes; ^2^ Tetraploid fishes.

In mammals, amphibians, reptiles, birds, sarcopterygii, and chondrichthyes (although there were two *aanat* genes, one non-vertebrate *aanat*, and one vertebrate *aanat*, found in the elephant shark, in this paper we focused only on vertebrate type), only one *aanat* gene was identified in their genomes. However, in teleosts, there are several copies of *aanat* genes. For common diploid teleosts, there are two or three different *aanat* genes. In detail (Table 1 and Table 2), for species like zebrafish (*Danio rerio*) possessing two *aanat* genes, there is one *aanat1* expressed in the retina, and one *aanat2* expressed in the pineal gland. For those like medaka (*Oryzias latipes*) with three *aanat* genes, there are two *aanat1s,* which have subtypes *aanat1a* and *aanat1b* expressed in the retina, and one *aanat2* in the pineal gland. Interestingly, for giant-fin mudskipper (PM) and Atlantic cod, there are *aanat1b* and *aanat2* with *aanat1a* missing. For a typical tetraploid teleost, like Atlantic salmon (*Salmo salar*) which experienced the salmonid-specific genome duplication, its genome has five copies of *aanat* genes, having lost one copy of its *aanat1a*. Likewise, in another tetraploid group, the golden-line fishes (SIA, *Sinocyclocheilus anshuiensis*; SIG, *Sinocyclocheilus graham*; SIR, *Sinocycloheilus rhinocerou*s), there are three *aanat* isoforms (compared to the expected six), due to loss of one *aanat1a* and both *aanat1b*s.

### 2.2. The Phylogenetic Relationships among aanat Genes

**Figure 2:**
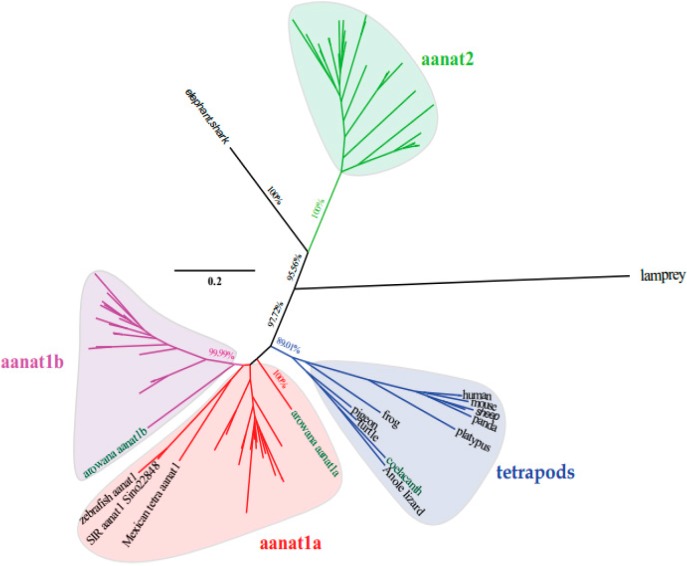
The radial Bayesian phylogenetic tree based on the conserved blocks of all *aanat* CDS sequences. The analysis was performed using the MrBayse 3.2 software. Evolutionary model selection was calculated using the MrMTgui program (the best nucleotide substitution model was GTR + I + G). The phylogenetic tree is rooted with lamprey (*Petromyzon marinus*) *aanat*. Although the PhyML and Mega phylogenetic trees are not shown here, they present the same tree topology. Four *aanat* subgroups are shown with different colors (Blue, tetrapod *aanat*; Red, teleost *aanat1a*; Magenta, teleost *aanat1b*; Lime, teleost *aanat2*).

**Figure 3:**
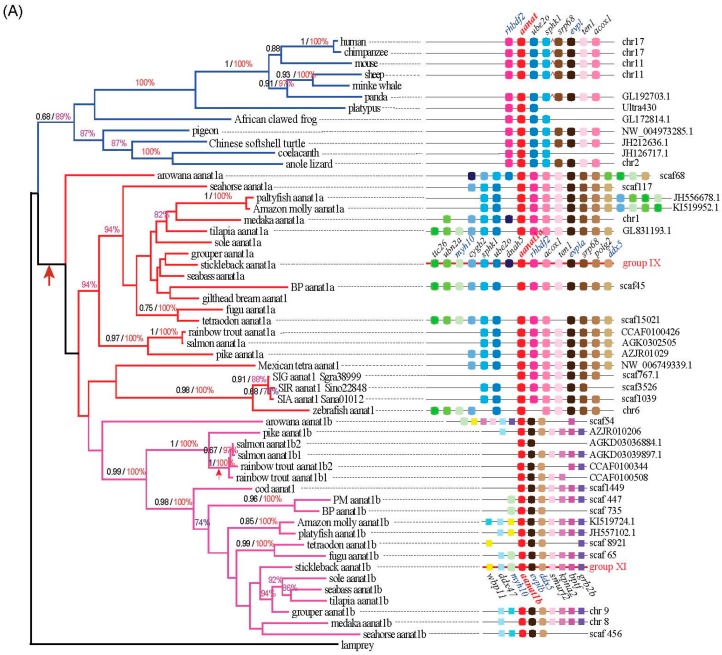

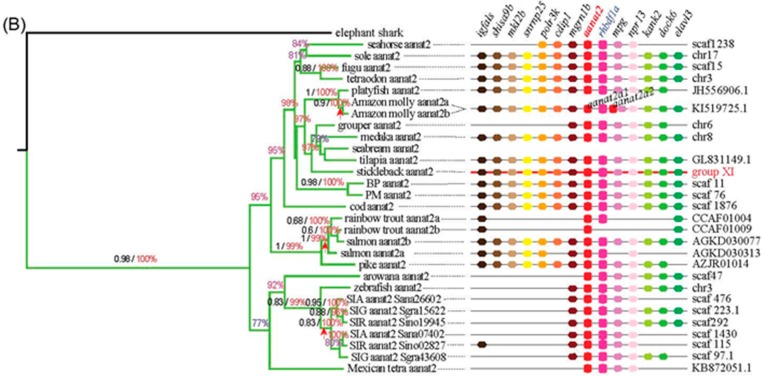
Phylogenetic trees and synteny of *aanat1* (**A**) and *aanat2* (**B**). The **left** of (**A**,**B**) are rectangular Bayesian trees of *aanat1* and *aanat2*, respectively. Numbers on branches from left to right are bootstrap supports (black) obtained in the phyML reconstruction and the Bayesian posterior probabilities (colored). The symbol of arrow represents the whole genome duplication. Note that bootstrap support under 0.6 and posterior probabilities under 70% are not shown. The color of branches is similar to Figure 2; The **right** of (**A**,**B**) are synteny of *aanat1* and *aanat2*, respectively. Distances between genes or gene length are not drawn to scale. In mammals, four genes (*srp68, evpl, ten1, acox1*) are located 400-kb upstream of *aanat* within several genes intervening (shown with a red broken line). Stickleback *aanat1a*, shown with a red backbone, is used as the reference sequence.

**Figure 4:**
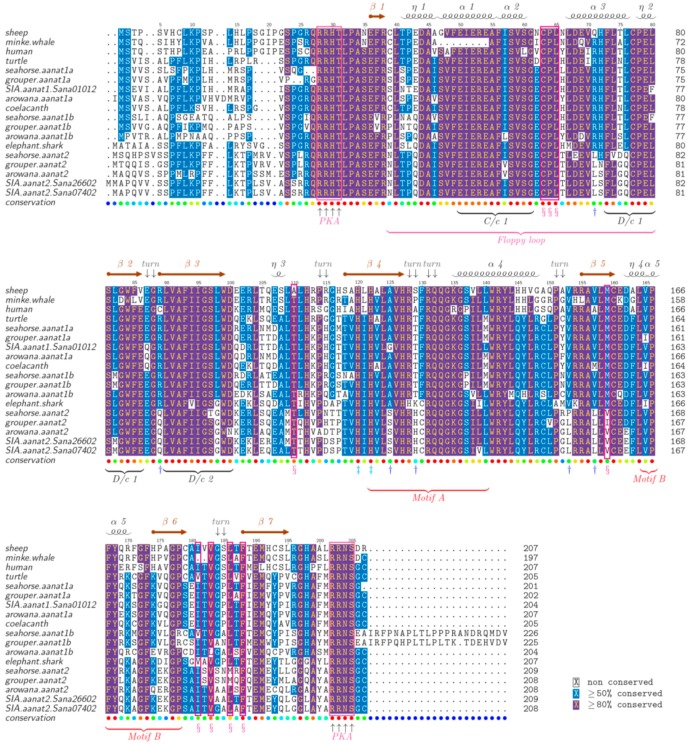
Alignment of AANAT protein sequences. AANAT proteins from different species were aligned with sheep AANAT and the structural template 1CJW. The analysis was completed with MAFFT and colorized using TEXshade. Secondary structures include alpha helix (α), beta strand (β), and 3^10^ helix (η). Important residues, including substrate binding residues (§, red boxes), catalytic residues (‡) and phosphorylation sites (up arrow, pink boxes), are marked for comparison. Residues differentiating between AANAT1 and AANAT2 are marked by a dagger (†). Numbering is relative to sheep AANAT. The color code for the conservation track ranges from red for the most conserved, to blue for the least conserved, as per TEXshade.

Using the lamprey (*Petromyzon marinus*) *aanat* as the out-group, three different reconstruction methods of phylogenetic trees generated the same tree topology with slight differences. According to the conserved blocks of *aanat* coding sequence (CDS), we generated the radial Bayesian tree in Figure 2. The left of Figure 3A is the *aanat1* rectangular Bayesian tree and the left of Figure 3B is the *aanat2* phylogenetic tree. According to the phylogenetic tree, the *aanat* gene divides into two main groups (*aanat1* and *aanat2*). Specifically, teleost *aanat1* is much closer to the tetrapod *aanat*, while *aanat2* is more distant and seems to be teleost unique. Teleost *aanat1* divides into two subgroups (*aanat1a* and *aanat1b*) and separates from tetrapod *aanat*. Interestingly, in the ancient fish Asian arowana (*Scleropages formosus*) *aanat1a* locates out of the entire teleost *aanat1* clade. In another ancient fish coelacanth (*Latimeria chalumnae*) the single *aanat* gene locates within the reptile clade, represented here by the Anole lizard (*Anolis carolinensis*). Mexican tetra (*Astyanax mexicanus*), three kinds of golden-line fishes (SIA, SIG, and SIR) and zebrafish belong to Ostariophysi, and in contrast to other teleosts possess only a single copy of the *aanat1* gene; this gene seems to be more closely related to *aanat1a* in the phylogenetic analysis shown in Figure 2, but related to *aanat1b* in the left panel of Figure 3a. Ultimately, synteny analysis (Figure 3a) and protein sequence alignment (Figure 4) confirm that the Ostariophysi *aanat1* corresponds to *aanat1a*. Within these same five Ostariophysi species, their *aanat2* seems to be divided from other teleost *aanat2* and to form a new clade. Protacanthopterygii (Atlantic salmon; rainbow trout, *Oncorhynchus mykiss*) *aanat1b* as well as *aanat2* diverge and form two different clades, consistent with the Salmonidae experiencing an additional unique whole genome duplication.

### 2.3. Synteny

All *aanat* genes share a conserved suite of genes bounding them on their side, although some species may display gene loss and location variation (see details in the right of Figure 3A,B). Seven genes (*rhbdf2*, *ube2o*, *sphk1*, *srp68*, *evpl*, *ten1*, and *acox1*) are neighboring the tetrapod *aanat* gene. In mammals, four genes (*srp68*, *evpl*, *ten1*, and *acox1*) are located 400-kb upstream of *aanat* within several intervening genes. All these neighboring genes can be identified in fish genomes with minor variations in location. *Rhbdf* can be found adjacent to *aanat1a* and *aana2*, but not *aanat1b*. *Evpl* can be found in tetrapod *aanat* and teleost *aanat1* regions. Three genes (*myh10*, *myh11b*, and *ddx47*) and six genes (*evp1b*, *ddx5*, *smurf2*, *kpna2*, *bptf*, and *grb2b*) are located upstream and downstream, respectively, of fish *aanat1b* genes. Two genes (*ddx5* and *evpl/evpl1b*) appear near the two isoforms of *aanat1*. Seven genes (*igfals*, *shisa9b*, *mkl2b*, *snrnp25*, *polrk3k*, *cdip1*, and *mgrn1b*) are located upstream of fish *aanat2*; six genes (*rhbdf1a*, *mpg*, *nprl3*, *kank2*, *dock6*, and *elavl3*) are positioned downstream of fish *aanat2*. The gene context of the two copies of Amazon molly *aanat2* shows differences because these two copies of *aanat2* are close to each other and locate within the same scaffold. Ostariophysi, compared with other teleosts, has lost many genes near *aanats*. All data illustrate that the synteny regions were conserved across species with slight differences.

### 2.4. The Regulatory Elements of aanat Genes

**Figure 5:**
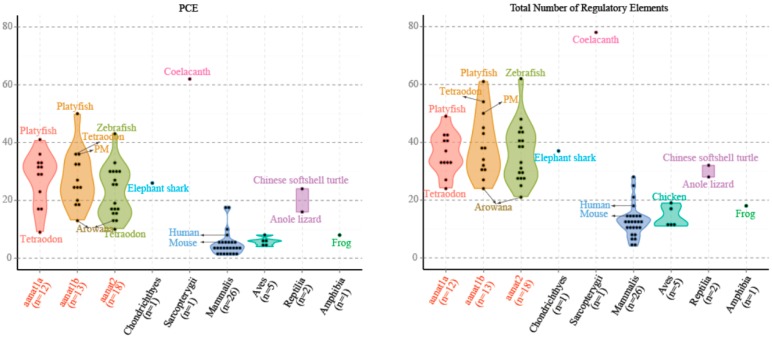
The numbers of PCE (Photoreceptor Conserved Elements) and the total numbers of regulatory elements (PCE, E-box, E-box-like, and PIPES, as defined in the Introduction) for different classes of *aanat*. We count *aanat* regulatory elements from 54 species, including those species mentioned in Supplementary Figure S1. The average number of *aanat* regulatory elements in fishes is larger than that of non-fish species.

In mammals, such as human and mouse, there are fewer PCEs associated with *aanats* compared to fishes (Figure 5 and Supplementary Figure S1). This is correlated with a significantly reduced role for photoreception in control of *aanat* expression in mammalian pinealocytes compared with their teleost counterparts. The total number of E-box and E-box-like elements are seven in human and eight in mouse. In contrast, the least is one in Mexican tetra *aanat1* and Atlantic salmon *aanat1b2*, and the most is 14 in fugu *aanat2* because of the short tandem repeats. The average number of E-box and E-box-like elements for teleost *aanat2* is much more than that of teleost *aanat1.* This suggests that E-box and E-box-like elements are the likely key regulators of pineal-specific *aanat* expression.

### 2.5. The Structure of AANAT Proteins

Representative AANAT proteins are aligned in Figure 4. The similarity and identity of these AANAT proteins are summarized in Supplementary Table S1. Previous research [12,30] has shown that AANAT possesses a catalytic funnel to bond with the reactive groups (*i.e*., the amine and AcCoA). Catalytic histidines (His_120_, His_122_) at the bottom of this pocket form a proton channel that deprotonates the protonated amine group to initiate the acetyl transfer reaction. The thioester bond of AcCoA comes under nucleophilic attack through the deprotonation. A conserved tyrosine (Tyr_168_) promotes protonation of the CoA thiolate-leaving group.

As shown in this alignment, amino acid residues 130 and 153 clearly differentiate between AANAT1 and AANAT2, regardless of species. Position of 130-amino acid of AANAT1 is Phe (F) compare with the Cys_130_ of AANAT2. The position of 153-amino acid of AANAT1 is Val (V) compared with the Leu_153_ of AANAT2. According to the template 1CJW [31], 153-amino acid locates close to the β-turn and the 130-amino acid participates in forming a turn. Therefore, F/C variation may play the biggest difference of these two kinds of AANATs. Some other variation sites also are pointed out in Figure 4. Altogether, our alignments show the highly conserved nature of AANAT proteins in all vertebrates.

## 3. Discussion

This study surveys a range of facets of the *aanat* gene and provides a global view about the diversity of AANAT in fish, not only from the DNA level but also from the protein level. We compared not only the differences in the regulatory regions, but also protein structural differences.

### 3.1. The Potential Reasons of Copy Number Variation among Vertebrates

Jack Falcon and his co-workers [14] clarified that 500 million years ago vertebrate *aanat* (*VT-aanat*) evolved from the invertebrate *aanat* (*NV-aanat*) isoform after the vertebrates split from the cephalochordates. *NV-aanat* is suggested to act as a detoxifier to prevent toxic reactions from endogenous and exogenous aralkylamines and polyamines [14]. The evolution of *VT-aanat* was facilitated by a duplication of *NV-aanat* early during vertebrate evolution. One duplicated copy was neofunctionalized to function primarily in the acetylation of serotonin in the synthesis of melatonin. Eventually the other copy, which would be recognized as the *NV-aanat* form, was lost from the vertebrate lineage.

It has been proven that two whole-genome duplications (WGD) occurred in the common ancestor of early vertebrates before ray-fined fishes split with tetrapods [32,33,34]. More specifically, the first round of WGD occurred before the agnatha-gnatostoma split and the second occurred before the chrondrichthyes-osteichthyes split. Subsequently, a third round of teleost-specific WGD occurred. Some fish families (Acipenseridae, Catostomidae, Cobitidae, Cyprininae, and Salmonidae) experienced a fourth round [35]. The teleost-specific WGD is thought of as the main evolutionary source of new genetic traits to support the expansion of this group. According to our analysis, we can confirm that the main reasons for copy number variation of *aanat* genes in different vertebrates are WGD and gene loss. The ancestral *VT-aanat* formed two copies of *aanat* gene (*aanat1* and *aanat2*) after undergoing WGD. The tetrapods and the ancient fishes, including the Actinistia coelacanth, lost aanat2 after they split with the bony fish, meanwhile the Holocephalan elephant shark lost aanat1. Because of another special WGD in teleosts, the teleost genomes may contain double copies of genes compared to their tetrapod counterparts. Hence, the teleost *aanat1* splits into two forms: *aanat1a* and *aanat1b*. Meanwhile, according to our synteny analysis, the two copies of *aanat2* may lose a copy and eventually form only one copy of *aanat2* gene in the teleost genomes.

The tetrapod and the ancient fish *aanat*s are similar to the bony fish *aanat1a* in the gene sequence (details shown in Figure 5 and Supplementary Table S1). The phylogenetic analysis also supports the view.

For tetraploid teleosts like rainbow trout [22,36], the salmonid-specific genome duplication leads to appearance of five copies of *aanat* genes in the genome. This phenomenon was also observed in Atlantic salmon. In Amazon molly, another tetraploid species, one copy of *aanat1a* and one copy of *aanat1b* are lost, generating four *aanat* genes in its genome. However, synteny analysis of Amazon molly indicates that the existence of two *aanat2* is due to a tandem duplication, not because of genome duplication. The copy number between diploid and tetraploid is not always the one-to-two correspondence due to selective gene loss.

With the development of new sequencing technology, the quality of sequenced whole genomes has been vastly improved. We therefore can gain more complete information about *aanat* gene in each genome. For example, we originally thought that there were only two copies of *aanat* genes existing in European seabass (*Dicentrarchus labrax*), which were *aanat1a* and *aanat1b*. However, now *aanat2* has been also identified using the newest version of European seabass reference genome [37]. More accurate and complete information about the *aanat* genes may be obtained in the future, and the evolution of *aanat* in fish can be better illustrated.

### 3.2. Adaptive Evolution of aanat

It is interesting that teleosts have retained multiple isoforms of AANAT where chondrichthyes and tetrapods have retained only one. This implies that there is some selective advantage to having multiple forms. In addition to having a spatial specialization, with AANAT1 being expressed primarily in the retina and AANAT2 being expressed primarily in the pineal gland, there are also instances where different isoforms are temporally regulated within a tissue. For example, two isoforms of *aanat1* (ss*aanat1a* and ss*aanat1b*) from the metamorphic flatfish Senegalese sole retina have been identified [38]. The timing of expression of these two *aanat1*s differs during sole development. In the early stages, ss*aanat1a* increases, reaching a peak at 4 dpf (days post fertilization) with a clear daily rhythm; after that its expression declines, and becomes arrhythmic. In contrast, ss*aanat1b* increases after metamorphosis, with a day/night rhythm in the adult. These data suggest different roles for the two ss*aanat1* during metamorphosis.

An instance where loss of an AANAT1 isoform may have functional consequences may be found in amphibious mudskippers [39]. Fully aquatic fish would be expected to have myopic vision out of water, but amphibious mudskippers apparently do not have this problem. AANAT1a in the retina can acetylate dopamine [26], and low-levels of dopamine during development may result in myopia [40]. As a consequence You *et al.* [39] speculated that loss of *aanat1a* in the genome of terrestrial giant-fin mudskipper compared with water-living blue-spotted mudskipper, may be adaptive for its aerial vision. Mexican tetra (*Astyanax mexicanus*), three kinds of golden-line fishes (SIA, SIG, and SIR) and zebrafish belong to Ostariophysi, and in contrast to other teleosts possess only a single copy of the *aanat1* gene; this gene seems to be more closely related to *aanat1a* in the phylogenetic analysis shown in Figure 2, but related to *aanat1b* in the left panel of Figure 3a. Ultimately, synteny analysis (Figure 3a) and protein sequence alignment (Figure 5) confirm that the Ostariophysi *aanat1* corresponds to *aanat1a*. Within these same five Ostariophysi species, their *aanat2* seems to be divided from other teleost *aanat2* and to form a new clade. However, the functional significance of these two isoforms of *aanat1* in teleosts is still under investigation.

The principle function of teleost *aanat2* appears to be melatonin synthesis. Like tetrapod *aanat*, teleost *aanat2* is primarily expressed in the pineal gland. The kinetics of teleost *aanat2* is similar to that of tetrapod *aanat* in terms of substrate selectivity [22]. Intriguingly, it is teleost *aanat1* that is more closely phylogenetically related to tetrapod *aanat*. Apparently this is related to the duplicated copies of *aanat* present in the common ancestor of teleosts and tetrapods. Following divergence of these lineages, tetrapod *aanat* retained or acquired characteristics of what would become teleost *aanat2*. Meanwhile, teleost *aanat1* evolved to gain traits specific for functions related to the retina. A better understanding of the ancestral forms of *aanat* present at the point of divergence would help decipher this dilemma.

## 4. Experimental Section

### 4.1. Sequence Collection

For our research, *aanat* nucleotide and amino acid sequences were collected from 37 species for comparison. For details, those reported *aanat* sequences, for example human (*Homo sapiens*) and zebrafish (*Danio rerio*), were downloaded from NCBI and Ensembl (Table 1). AANAT sequences of Malabar grouper (*Epinephelus malabaricus*), half-smooth tongue sole (*Cynoglossus semilaevis*), giant-fin mudskipper or PM (*Periophthalmus magnuspinnatus*), blue-spotted mudskipper or BP (*Boleophthalmus pectinirosris*), seahorse (*Hippocampus spinosissimus*), Atlantic salmon (*Salmo salar*), rainbow trout (*Oncorhynchus mykiss*), pike (*Esox lucius*), golded-line fishes (SIA, *Sinocyclocheilus anshuiensis*; SIG, *Sinocyclocheilus graham*; SIR, *Sinocyclooheilus rhinocerous*), Asian arowana (*Scleropages formosus*), and Mexican tetra (*Astyanax mexicanus*) were extracted from the genome databases, generated by us and our collaborators, through BLAST [41] and Gene Wise [42].

### 4.2. Sequence Alignment and Phylogenetic Reconstruction

We conducted phylogenetic analyses using these collected sequences. After finishing the alignment of *aanat* gene sequences using MAFFT software [43], we chose conserved blocks by the online Gblocks [44]. Subsequently, MrMTgui program [45] was applied to complete the nucleotide substitution model test (the best model based on the Akaike Information Criterion, AIC, was GTR + I + G). Finally, we analyzed the sequences using MrBayes 3.2 [46] through the Bayesian tactics with the Metropolis-coupled Markov chain Monte Carlo algorithm [47]. By using four chains run, setting four million generations for Ngen and 100 for Samplefreq, the best phylogenetic tree was explored. The first 5000 trees were discarded (Burnin) before calculating summary statistics that allowed identification of the point when the chains became fixable. A consensus tree was constructed with the remaining trees. Branch support was evaluated using Bayesian posterior probabilities.

In addition, PhyML [48] and MEGA 6 [49] were used to rebuild AANAT protein phylogenetic trees, with a local Prottest [50] program to analyze the amino acid substitution model (the best substitution model was JTT + G + F) and the 1000 bootstrap replications. The best tree was explored using PhyML and MEGA 6 with manual alignment corrections.

Meanwhile, we also selected the tightly conserved mitochondrial gene *cytb* of the examined species to build the species phylogenetic tree using the same methods. Comparing the species phylogenetic tree and the AANAT tree, we evaluated the relatedness between species evolution and *aanat* gene evolution.

### 4.3. Identification of Conserved Synteny

To assess the degree of *aanat* gene conservation, we searched several genes upstream and downstream of each *aanat* paralog. We collected genome data from Ensembl dataset and our lab as mentioned in Section 2.1. The stickleback (*Gasterosteus aculeatus*) genome was used as the reference sequence for searching *aanat* upstream and downstream. The genome assemblies of different species were searched using the BLAST and the best hit was selected using a Perl script.

### 4.4. Analysis of Regulatory Regions and the Differences between AANAT Proteins

We obtained the complete genomic sequences of including 4 kb upstream and downstream of 84 *aanat* genes. Four typical regulatory elements, including E-box, E-box like element, PCE and *cis*-acting regulatory element, were searched for in each sequence including the upstream and downstream regions using BLAST program. The similarity of target was required to be 100% with the sequences of four regulatory elements (described in Introduction Section).

We also downloaded a common AANAT protein model of sheep (*Ovis aries*, 1CJW) from the Protein Data Bank (PDB) for comparing the difference between AANAT1 and AANT2. MAFFT software was used to complete alignment. The result of alignment was colorized using TEXshade [51].

## 5. Conclusions

The diversity of *aanat* in fish is generated by whole genome duplication and gene loss. During evolution, due to different external circumstances, *aanat1* and *aanat2* separated, were expressed in different organs and took on different roles. In this paper, we have investigated and refined this view based on additional sequences from various fish species not previously available. At the same time, we also use an evolutionary view to reveal many similarities or identities between AANAT1 protein and AANAT2 protein. Through these analyses, to some extent, the evolutionary features of *aanat* also reveal aspects of the relationship between the pineal gland and the environmental circumstance.

## Supplementary Materials

Supplementary materials can be found at http://www.mdpi.com/1422-0067/17/1/51/s1.

## Abbreviations

*acox1:* peroxisomal acyl-coenzyme A oxidase 1; *bptf:* nucleosome-remodeling factor subunit BPTF; *cdip1*: cell death-inducing p53 target 1; *cygb2*: cytoglobin 2; ddx47: DEAD (Asp-Glu-Ala-Asp) box polypeptide 47; *ddx5*: DEAD (Asp-Glu-Ala-Asp) box polypeptide 5; *dock6*: dedicator of cytokinesis 6; *elavl3*: ELAV like neuron-specific RNA binding protein 3; *evpl*: envoplakin; *evpla*: envoplakin a; *evplb*: envoplakin b; *grb2b*: growth factor receptor-bound protein 2b; *igfals*: insulin-like growth factor binding protein, acid labile subunit; *kank2*: KN motif and ankyrin repeat domains 2; *kpna2*: karyopherin alpha 2; *mgrn1b*: MGRN1 mahogunin ring finger 1, E3 ubiquitin protein ligase; *mkl2b*: MKL/myocardin-like 2b; *mpg*: DNA-3-methyladenine glycosylase; *myh10*: myosin, heavy chain 10, non-muscle; *nprl3*: nitrogen permease regulator-like 3; *polg2*: polymerase (DNA directed), gamma 2; *polr3k*: polymerase (RNA) III (DNA directed) polypeptide K; *rhbdf1a*: rhomboid 5 homolog 1a; *rhbdf2*: inactive rhomboid protein 2; *shisa9b*: shisa family member 9b; *smurf2*: SMAD specific E3 ubiquitin protein ligase 2; *snrnp25*: small nuclear ribonucleoprotein 25; *sphk1*: sphingosine kinase 1; *srp68*: signal recognition particle 68; *ten1*: TEN1 CST complex subunit; ttc26: tetratricopeptide repeat domain 26; *ube2o*: ubiquitin-conjugating enzyme E2 O; *ubn2a*: ubinuclein 2a; *wbp11*: WW domain-binding protein 11.
