# Supplementary material for: Molecular Evolution of Aralkylamine N-Acetyltransferase in Fish: A Genomic Survey
Source: Int J Mol Sci. 2015 Dec 31;17(1):51. doi: 10.3390/ijms17010051 (PMC4730296; doi:10.3390/ijms17010051)
Supplement: Supplementary file 1 [file ijms-17-00051-s001.pdf]

Supplementary Materials: Molecular Evolution of Aralkylamine *N*-Acetyltransferase in Fish: A Genomic Survey

Jia Li, Xinxin You, Chao Bian, Hui Yu, Steven L. Coon and Qiong Shi

Table S1. The similarity and identity of 18 AANAT proteins used in Figure 5 for alignment of AANAT protein sequences.

Species name	Sheep	Minke whale	Human	Turtle	Seahorse aanat1a	Grouper aanat1a	SIA aanat1 Sana01012	Arowana aanat1a	Coelacanth	Seahorse aanat1b	Grouper aanat1b	Arowana aanat1b	Elephant shark	Seahorse aanat2	Grouper aanat2	Arowana aanat2	SIA aanat2 Sana26602	SIA aanat2 Sana07402
Sheep	—	88.3	81.6	79.9	77.5	77.1	77.8	78.8	79.9	73.8	74.8	78.8	74.1	69.9	69.4	70.8	70.8	70.8
Minke whale	82.7	—	82.2	78.3	75.7	77.4	76.6	78.2	78.8	70.9	73.5	76.1	74.3	68.8	69.3	70.4	70.9	70.9
Human	77.2	76.6	—	76.4	76.5	75.1	75.8	77.8	76.9	73.3	74.3	75.8	74.1	69.9	69.4	72.8	70.8	70.8
Turtle	69.6	69	69.6	—	87.5	90.5	88.2	89.7	92.1	81.3	86.2	83.8	83.4	80.4	79	79	78.5	79
Seahorse aanat1a	68.5	66.3	68	81.5	—	94.4	93	94	92	80.5	85.5	83.5	83	79.6	79.6	79.1	80	79.6
Grouper aanat1a	66.6	65.9	66.1	82.6	92.4	—	94.5	93.5	93.5	80.6	87.1	84.1	82.6	81.1	80.6	79.7	79.2	79.7
SIA.aanat1 Sana01012	67.4	65.8	66.5	80.8	88	88.1	—	92.6	92.6	82.8	88.2	83.3	83.8	78.9	79.4	77.9	79.4	78.9
Arowana aanat1a	67.9	67.3	69.4	83.3	91	89.1	88.7	—	93.6	80.3	86.2	84.8	83.9	80.9	80.4	79.5	80	79.5
Coelacanth	68.6	67	66.6	86.3	88	89.1	87.2	89.2	—	80.8	85.7	85.7	84.8	81.4	80.9	79.5	79.5	79.5
Seahorse aanat1b	64	61.6	63	74.5	75.1	75.2	76.4	75.9	73	—	88.8	78.9	77.9	72.5	72.5	71.5	72.5	72
Grouper aanat1b	64.5	62.1	64	77.4	80.5	81.6	80.8	79.9	78.9	84	—	80.8	80.3	76.9	76.9	74	74.5	74
Arowana aanat1b	66	65.8	64	75.4	75.1	73.7	73	75.9	75.4	71	71	—	75.4	75	74	76.9	75	75
Elephant shark	61.9	61.5	60.4	72.6	72.6	72.2	72.5	71.7	73.6	66.1	69.1	64.7	—	86.4	85	84	86.9	86.9
Seahorse aanat2	60.6	58.6	59.7	70.2	69.6	69.8	69.1	69.7	70.2	63.2	67.1	62.7	72.4	—	94.2	88.9	89.4	89.4
Grouper aanat2	59.7	58.6	59.2	69.2	69.1	69.3	68.6	69.2	69.7	62.2	66.1	62.7	72.9	89.4	—	89.9	90.8	90.8
Arowana aanat2	60.1	59.1	60.6	67.3	67.6	67.8	67.1	66.8	67.8	62.2	64.7	65.1	70	81.7	84.1	—	92.3	92.7
SIA aanat2 Sana26602	60.1	59.6	59.2	68.2	70.1	68.8	69.6	68.2	70.2	64.2	67.6	63.7	74.8	83.1	84.6	87.9	—	99
SIA aanat2 Sana07402	60.6	60.2	59.7	69.2	70.1	69.8	69.6	68.2	70.7	64.2	66.6	64.2	75.8	82.6	84.1	87	97.5	—

% Identity

% Similarity

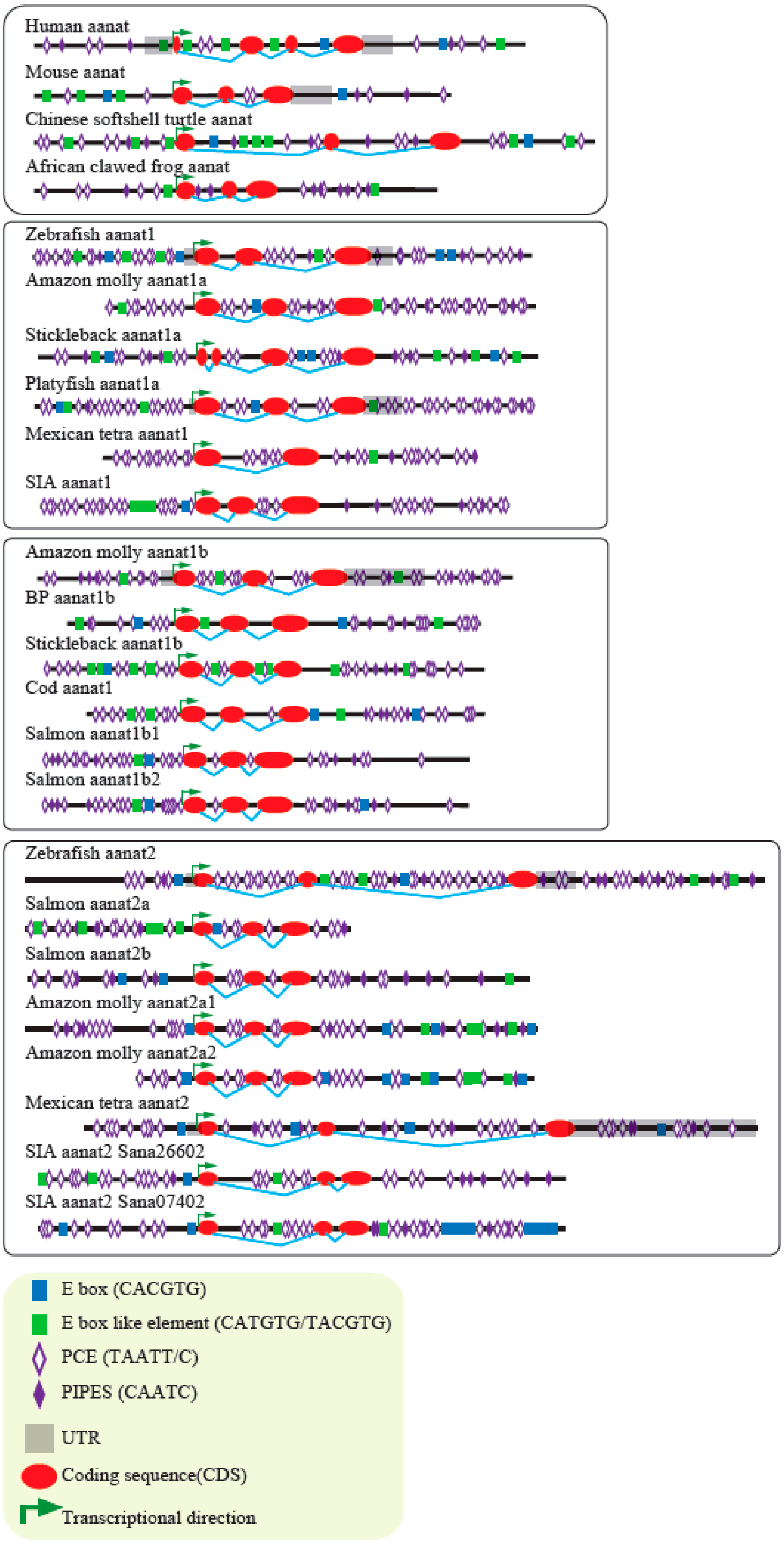

Figure S1. Schematic representation of typical regulatory elements for different *aanat* genes.
